# Bridging the Gap in Undergraduate Radiology Education: A Literature Review and Prospective Evaluation of Case-Based Teaching

**DOI:** 10.7759/cureus.110966

**Published:** 2026-06-16

**Authors:** Furkan Zurel, Ryan Pathak

**Affiliations:** 1 Radiology, Salford Royal NHS Foundation Trust, Salford, GBR; 2 Research, The University of Manchester, Manchester, GBR

**Keywords:** case-based learning, curriculum development, educational intervention, imaging interpretation, medical education, pilot radiology teaching, radiology, radiology education, radiology teaching, undergraduate curriculum

## Abstract

Background

Radiology is fundamental to modern medicine, yet undergraduate exposure remains variable. Inconsistent curricula and reliance on traditional lectures risk leaving graduates underprepared to interpret imaging, make appropriate requests, and integrate radiology into patient care. This study aimed to review published undergraduate radiology curricula and teaching methods in the UK and internationally, explore Year 3 medical students’ understanding and perceptions following pilot radiology teaching sessions at the University of Manchester, and propose level-appropriate curriculum suggestions to address gaps.

Methods

A structured literature review was conducted across PubMed, Embase, and Google Scholar (2000-2025), encompassing studies that described undergraduate radiology curricula, teaching modalities, and assessment strategies. A prospective educational intervention was delivered to Year 3 medical students covering chest radiographs, CT head scans, imaging request justification, and imaging safety. Pre- and post-session surveys assessed confidence, knowledge of imaging safety, and radiology perceptions, alongside prior exposure to radiology teaching. Paired comparisons were analysed using paired t-tests and free-text responses underwent thematic analysis.

Results

UK and European consensus curricula emphasise core competencies for undergraduate radiology, including interpretation of common imaging (chest X-ray, acute CT head), safe imaging practices, and effective communication of findings. Delivery is inconsistent across schools; foundation doctors are expected to request appropriate investigations and integrate imaging into clinical care. Twenty-eight students attended the pilot radiology teaching sessions, of whom 26 completed paired pre- and post-session surveys and comprised the analytic sample (response rate 92.9%). Most Year 3 students reported limited prior radiology teaching, variable understanding of the speciality, and underestimation of radiology’s role within the NHS. Mean self-reported confidence in chest radiograph interpretation increased from 3.1 ± 1.4 to 7.6 ± 1.2 (mean difference 4.5, 95% CI 3.9-5.1; p < 0.001). Confidence in acute CT head interpretation increased from 2.8 ± 1.3 to 7.2 ± 1.1 (mean difference 4.4, 95% CI 3.8-5.0; p < 0.001). Awareness of radiation dose differences improved from 38% to 81%. Most students reported limited prior radiology teaching, while 88% preferred case-based teaching and 85% supported earlier, clinically integrated radiology exposure in the curriculum.

Conclusion

Undergraduate radiology education remains heterogeneous and often insufficient to meet early postgraduate expectations. Evidence supports case-based, interactive approaches that align with clinical practice. To bridge this gap, our findings highlight a need for a structured, competency-based radiology curriculum in UK medical schools, with emphasis on early exposure, integration with clinical placements, and multimodal teaching.

## Introduction

Radiology underpins diagnosis and clinical management in almost every speciality. In the United Kingdom (UK), 45 million scans were reported in 2023, a rise compared to previous years [1]. Despite this, radiology often receives limited attention in undergraduate curricula, with many medical students and resident doctors reporting inadequate confidence in interpreting common imaging modalities [2,3].

The Royal College of Radiologists (RCR) has recommended a structured undergraduate curriculum emphasising image interpretation, justification of requests, and patient safety [4]. International consensus, including the European Society of Radiology (ESR), similarly calls for integration of radiology into anatomy, clinical teaching, and digital learning platforms [5]. Nevertheless, delivery remains inconsistent, with some medical schools offering only a few hours of radiology teaching across the entire medicine degree [6,7].

This variability risks leaving new doctors underprepared for clinical practice, particularly in high-stakes settings such as acute care, where early recognition of imaging abnormalities can alter patient outcomes. Addressing these gaps is essential to prepare students for early postgraduate practice, where safe imaging use and interpretation are core competencies. This study therefore aimed to provide contextual background through a literature review of undergraduate radiology curricula and teaching methods, and to deliver and evaluate a pilot case-based radiology teaching intervention for Year 3 medical students at the University of Manchester. The prospective educational evaluation represents the primary empirical component of the study.

## Materials and methods

Literature review

A narrative literature review was performed across PubMed, Embase, and Google Scholar, covering January 2000 to December 2025, to identify key publications describing undergraduate radiology curricula, teaching modalities, and assessment strategies. Search terms included ‘radiology’, ‘undergraduate’, ‘medical students’, ‘curriculum’, ‘early postgraduate’, and ‘teaching’. Final searches were run on 07/12/2025.

Titles and abstracts were screened to identify studies describing undergraduate radiology curricula, educational interventions, and assessment approaches relevant to medical student training. Full texts were reviewed where appropriate to determine relevance to undergraduate radiology education. The database searches yielded 118 records. Following removal of duplicates and title/abstract screening, 42 articles underwent full-text review. Twenty-five publications were considered directly relevant to undergraduate radiology education and were included in the final literature review. A representative PubMed search strategy was: ("radiology" AND "undergraduate" AND ("medical students" OR "medical education") AND ("curriculum" OR "teaching")). Screening and study selection were performed by the primary investigator.

As the purpose of this review was to provide contextual background for the pilot educational intervention rather than to perform a systematic evidence synthesis, no formal risk-of-bias assessment was conducted.

Inclusion Criteria

English-language studies; undergraduate radiology education (with early postgraduate studies included if clearly UG-relevant); and description of curriculum frameworks, teaching interventions, or assessment methods were included.

Exclusion Criteria

Purely postgraduate/specialty training studies, editorials without substantive data, and studies focused only on passing exams were excluded.

Data Extracted

Country/setting, learner year, recommended competencies, clinical expectations, teaching format, contact hours, curriculum framework, assessment type, and outcomes (knowledge, skills, confidence) were extracted. Findings were synthesised narratively under four headings: (i) standards and curricula, (ii) teaching modalities and effectiveness, (iii) assessment strategies, (iv) gaps/opportunities.

The purpose of this review was to provide contextual background for the pilot educational intervention rather than to perform a comprehensive systematic review of all undergraduate radiology curricula. Findings from the literature informed the design of pilot radiology teaching sessions delivered locally.

Pilot radiology teaching sessions and surveys

Two interactive, case-based teaching sessions, each lasting approximately 60 minutes, were delivered to Year 3 medical students at the University of Manchester in November 2025. The sessions were supervised by a consultant radiologist with experience in undergraduate radiology education. The teaching content was aligned with the RCR's undergraduate curriculum recommendations and introduced radiology as a clinical speciality, including its role within the NHS and multidisciplinary patient care pathways. Students were taught a systematic approach to chest radiograph interpretation, recognition of acute abnormalities on CT head imaging, and principles of imaging safety, including appropriate investigation requesting and justification.

Teaching content was standardised across sessions using a predefined slide set and case examples aligned with RCR undergraduate curriculum recommendations.

Inclusion criteria for the pilot educational intervention comprised Year 3 medical students enrolled at the University of Manchester who attended the radiology teaching sessions and consented to complete the pre- and post-session surveys. Students were eligible regardless of prior radiology teaching exposure or intended career interests. Exclusion criteria included students from other year groups, particularly pre-clinical students, those who did not attend the teaching sessions in full, and incomplete or unmatched survey responses that prevented paired analysis.

Students completed an online survey immediately before the teaching session (pre-session) and the same questionnaire immediately after the session (post-session) to enable paired comparisons. The questionnaire was developed by the authors to evaluate key educational domains relevant to undergraduate radiology training, including prior exposure to radiology teaching, confidence in interpreting common imaging studies, knowledge of imaging safety principles, and perceptions of radiology as a clinical speciality. Survey items were informed by published literature on undergraduate radiology education and by curriculum expectations outlined by the RCR. The questionnaire was reviewed internally by the supervising consultant radiologist prior to distribution to ensure clarity, relevance, and alignment with the learning objectives of the teaching sessions. The full questionnaire is provided in the Appendices.

The survey evaluated students’ prior exposure to radiology teaching, self-reported confidence in imaging interpretation before and after the intervention, knowledge of imaging safety principles, perceptions of the teaching session design, preferences for future radiology teaching, and understanding of radiology’s clinical and consultative role within the NHS. Confidence was rated on a 10-point numerical scale (1 = no confidence, 10 = very confident), while perceptions used 5-point Likert scales. Responses also included multiple-choice questions and free-text items.

Measures relating to students’ understanding of radiology were derived from questionnaire items exploring perceptions of radiology as a speciality, awareness of radiologists’ day-to-day responsibilities, recognition of radiologist involvement in multidisciplinary team working, and understanding of radiology’s role within NHS patient pathways. Responses indicating uncertainty regarding these aspects were classified as demonstrating limited awareness or appreciation of radiology’s broader clinical role. The specific survey items used to derive these outcomes are provided in the Appendices.

Statistical analysis

Statistical analysis was performed using paired t-tests to compare pre- and post-session self-reported confidence scores. Confidence ratings were measured using a 10-point numerical scale and analysed as approximately continuous variables, and paired t-tests were selected to assess within-participant changes following the educational intervention. This approach is commonly adopted in educational research when evaluating within-participant changes in confidence ratings. Visual inspection of the distribution of paired differences demonstrated no marked departures from normality, supporting use of parametric testing. Mean differences and 95% confidence intervals (CIs) were calculated to aid interpretation of the magnitude of observed changes following the educational intervention.

Descriptive statistics were used to summarise categorical survey responses, including prior exposure to radiology teaching, perceptions of radiology, preferences for future teaching, and changes in knowledge-based outcomes. Knowledge-based outcomes were scored as correct or incorrect according to predefined answers relating to imaging safety principles. Missing responses were excluded from calculation of the relevant percentage outcome. Paired contingency data required for formal paired categorical analyses were not retained as part of the original educational evaluation, and therefore knowledge-based outcomes are reported descriptively.

Free-text responses were analysed using thematic analysis. Responses were reviewed by the primary investigator and examined iteratively to identify recurring concepts and patterns. Similar responses were grouped into broader themes, which were refined to generate the final thematic categories reported. As the qualitative component was intended to provide descriptive insight within a pilot educational evaluation, formal independent coding and inter-rater reliability assessment were not performed. A p-value of < 0.05 was considered statistically significant.

Participation and ethical considerations

Participation in the survey was voluntary, and students were informed that participation was optional and would not affect their academic assessment. However, the survey was distributed immediately before and after scheduled teaching sessions, which likely contributed to the high response rate.

This study was conducted as an evaluation of undergraduate radiology teaching and was considered an educational quality improvement initiative. Survey responses were collected anonymously, and no identifiable personal data were obtained. Formal research ethics committee approval was not required.

## Results

Teaching session and survey results (University of Manchester)

Participant characteristics are summarised in Table 1.

**Table 1:** Participant characteristics. Unless otherwise stated, percentages reported in subsequent analyses are based on the 26 students who completed paired pre- and post-session surveys.

Characteristic	Value
Total attendees	28
Completed paired surveys	26
Response rate	92.9%

Key Insights

Key insights from the survey demonstrated limited understanding of radiology’s broader clinical, consultative, and decision-making roles among many students, based on questionnaire responses exploring awareness of radiologists’ day-to-day responsibilities, involvement in multidisciplinary team working, and radiology’s role within NHS patient pathways. Strong support was also expressed for earlier and more structured radiology teaching. A summary of key survey findings is presented in Table 2.

**Table 2:** Summary of students’ perceptions and understanding of radiology among participants who completed paired surveys (n = 26).

Survey finding	n (%)
Limited appreciation of radiology’s clinical and consultative role	16 (62)
Unsure of a radiologist’s day-to-day responsibilities	19 (73)
Recognised routine radiologist participation in multidisciplinary team meetings	6 (23)
Limited awareness of radiology’s role within NHS patient pathways and acute care	17 (65)
Supported earlier and more structured radiology teaching across both pre-clinical and clinical years	22 (85)

Thematic analysis of free-text responses highlighted a strong demand for hands-on, clinically relevant teaching and contextualisation of learning to prepare for postgraduate years.

Mean self-reported confidence in chest radiograph interpretation increased from 3.1 ± 1.4 to 7.6 ± 1.2 (mean difference 4.5, 95% CI 3.9-5.1; p < 0.001). Confidence in acute CT head interpretation increased from 2.8 ± 1.3 to 7.2 ± 1.1 (mean difference 4.4, 95% CI 3.8-5.0; p < 0.001). Observed awareness of radiation dose differences improved from 38% to 81%. These results demonstrate statistically significant increases in self-reported confidence across all measured confidence domains following the intervention. In addition, observed increases were seen in knowledge-based imaging safety outcomes; however, these findings are reported descriptively and should be interpreted accordingly. However, these measures reflect perceived confidence rather than objectively assessed diagnostic competence. Changes in self-reported confidence before and after the intervention are summarised in Table 3.

**Table 3:** Changes in students’ self-reported confidence following the case-based radiology teaching intervention (mean ± SD; 10-point scale). CXR: chest radiograph

Task	Pre-session, mean ± SD	Post-session, mean ± SD	Mean difference (95% CI)	p-value
CXR interpretation	3.1 ± 1.4	7.6 ± 1.2	+4.5 (3.9-5.1)	<0.001
Acute CT head interpretation	2.8 ± 1.3	7.2 ± 1.1	+4.4 (3.8-5.0)	<0.001
Imaging request justification	3.4 ± 1.5	7.8 ± 1.0	+4.4 (3.8-5.0)	<0.001

Prior exposure to radiology teaching is summarised in Table 4.

**Table 4:** Prior exposure to radiology teaching among participants who completed paired surveys (n = 26).

Prior teaching modality	n (%)
Lectures only	15 (58)
Case-based teaching	5 (19)
Online modules	4 (15)
No prior radiology teaching	2 (8)

Changes in students’ radiology knowledge and imaging safety awareness before and after the teaching intervention are summarised in Table 5.

**Table 5:** Observed changes in students’ radiology knowledge and imaging safety awareness among participants who completed paired surveys (n = 26).

Knowledge domain	Pre-session, n (%)	Post-session, n (%)
Awareness of relative radiation dose differences	10 (38)	21 (81)
Recognition of contrast-associated risks	12 (46)	22 (85)

Student perceptions of the teaching sessions are summarised in Table 6.

**Table 6:** Student perceptions of the teaching sessions among participants who completed paired surveys (n = 26).

Survey response	n (%)
Agreed/strongly agreed confidence improved	24 (92)
Preferred case-based teaching over lectures	23 (88)
Supported earlier, clinically integrated radiology exposure in the curriculum	22 (85)

Key themes identified from qualitative (thematic) analysis of free-text responses are summarised in Table 7.

**Table 7:** Themes identified from qualitative (thematic) analysis of free-text responses.

Theme	Summary
Need for increased radiology exposure	Students reported insufficient undergraduate radiology teaching and requested additional sessions
Value of case-based learning	Authentic clinical cases were perceived as the most useful learning approach
Demand for digital learning resources	Students requested online modules, image banks, and self-directed learning resources

Overall, the pilot radiology teaching sessions were well received and demonstrated substantial improvements in student confidence, knowledge of imaging safety, and understanding of radiology’s clinical role. These findings suggest potential value in integrating structured, case-based radiology teaching earlier within the undergraduate medical curriculum.

## Discussion

Literature review

Curriculum Frameworks

In the UK, the RCR’s undergraduate curriculum recommends core competencies in chest radiograph (CXR) and abdominal radiograph (AXR) interpretation, CT head for acute settings, safe imaging requesting, radiation awareness, and communication [4]. Implementation across UK schools is inconsistent, with reported teaching hours ranging widely [8]. Early postgraduate doctors are generally expected to competently interpret common imaging, request appropriate investigations safely, communicate findings clearly, and understand radiology’s role within patient care and multidisciplinary workflows [4].

Internationally, the ESR calls for integration into anatomy and clinical teaching, early introduction of imaging appropriateness, and emphasis on digital tools [5]. Flipped classroom models, increasingly adopted in US curricula, have demonstrated tangible benefits in radiology education [9]. Although these innovations align with established pedagogical theories such as constructivism, contact hours remain limited. Key curriculum competencies identified across published undergraduate radiology curricula are summarised in Table 8.

**Table 8:** Core radiology competencies in published curricula [4,5,8,9]. RCR: Royal College of Radiologists; ESR: European Society of Radiology; CXR: chest radiograph; AXR: abdominal radiograph

Source	Core topics	Educational emphasis	Notes
RCR (UK)	CXR, AXR, CT head, imaging safety, appropriate imaging requests	Preparation for early postgraduate practice and safe imaging utilisation	Limited implementation data
ESR (Europe)	Integration of imaging with anatomy, imaging appropriateness, digital learning resources	Early exposure and integration across undergraduate curriculum	Consensus statement only
US schools	Imaging safety, case-based, flipped classroom models	Interactive and learner-centred approaches	Greater emphasis on digitalisation and blended learning strategies

Teaching Modalities and Effectiveness

Lectures remain common but show limited effectiveness in fostering long-term confidence [10]. In contrast, case-based learning (CBL) consistently improves interpretation skills and supports clinical integration [11]. E-learning modules offer flexibility and accessibility, enhancing short-term test performance, but risk low engagement without interactivity [12,13]. Simulation and flipped classroom approaches demonstrate notable gains in learner confidence and application, although the evidence base remains limited [14]. A summary of reported undergraduate radiology teaching modalities and educational approaches is presented in Table 9.

**Table 9:** Summary of teaching modalities and reported outcomes [10-14].

Modality	Evidence summary	Reported benefits	Educational basis	Limitations
Lectures	Widely used in undergraduate curricula	Low cost, efficient delivery of knowledge	Traditional didactic teaching	Passive learning and limited retention
Case-based learning	Strong evidence in education	Improves confidence and clinical reasoning	Constructivist learning and contextual learning	Requires faculty time and preparation
E-learning	Mixed evidence across studies	Flexible and scalable learning	Self-directed learning models	Risk of reduced engagement
Simulation/flipped classroom approaches	Emerging evidence	Higher learner engagement and application	Active learning and experiential learning	Resource-intensive

Assessment Strategies

Few schools employ a structured assessment of radiology competence. Where tested (e.g. objective structured clinical examination (OSCE) stations, image quizzes), gains were measurable but unclear whether linked to progression [15,16]. Lack of standardised assessment remains a major gap.

Gaps and Opportunities

There is considerable variability in curricula and limited radiology exposure across UK medical schools, with an over-reliance on lectures despite stronger evidence supporting interactive methods. Integration into clinical placements is minimal, and structured, competency-based assessment is often lacking. This highlights an opportunity to implement targeted, case-based modules within the undergraduate curriculum.

This literature review and pilot survey highlight a persistent gap between the undergraduate radiology experience of medical students and the expectations of early postgraduate doctors. The RCR’s undergraduate curriculum indicates that medical graduates are expected to competently interpret common imaging, request investigations appropriately, apply radiation safety principles, communicate findings effectively, and understand the workflow and multidisciplinary integration of radiology in patient care [4]. Consensus curricula from Europe also emphasise these core competencies; however, delivery remains heterogeneous, with substantial variability in teaching hours, clinical exposure, and assessment approaches [5-7]. Comprehensive comparisons between institutions are therefore limited.

Our local findings mirror these broader patterns. Survey findings following teaching sessions from Year 3 medical students at the University of Manchester reinforce these observations. Students have limited prior exposure and frequently perceive radiology narrowly, with 62% demonstrating limited appreciation for its broader clinical and consultative roles. Seventy-three per cent were unsure of a radiologist’s day-to-day responsibilities, and only a minority recognised their involvement in multidisciplinary decision-making. Awareness of radiology’s role within NHS patient pathways and acute care was limited in 65% of students, and 85% expressed a strong request for earlier, clinically integrated exposure. Self-reported confidence in interpreting chest X-rays and CT heads improved significantly following the pilot interactive, case-based radiology teaching sessions, and 88% preferred this approach over traditional lectures. However, these improvements reflect perceived confidence rather than objectively measured diagnostic competence. Thematic analysis further highlighted a need for hands-on, clinically relevant teaching and the provision of digital resources, such as image banks or online modules, to support self-directed learning and prepare students for postgraduate practice [12,13]. The use of paired pre- and post-session measures reduces between-participant variability and permits assessment of immediate within-participant changes following the intervention, ultimately strengthening internal validity. However, attribution of observed changes to the intervention remains limited by the absence of a control or comparator group.

Collectively, these findings demonstrate that undergraduate radiology education, while increasingly recognised as essential, remains insufficiently aligned with the competencies expected of early postgraduate doctors. Traditional lecture-based methods alone appear inadequate, whereas interactive, case-based, and blended approaches show promise in improving confidence, knowledge, and practical skills [10,11,14]. Higher proportions of students demonstrated awareness of imaging safety concepts following the pilot case-based radiology teaching intervention. These approaches are consistent with constructivist learning theory, in which learners actively construct knowledge through engagement with authentic clinical problems and contextualised case-based discussion [17,18]. There is a clear opportunity to implement level-appropriate curricula that integrate radiology more explicitly into pre-clinical anatomy and core clinical years, emphasising both technical interpretation skills and the professional, collaborative role of radiologists within the healthcare system.

These proposals are informed by findings from a local pilot evaluation and narrative literature review and should be considered areas for future investigation and multi-institutional evaluation rather than direct conclusions of the present study.

To translate these insights into tangible educational outcomes and ensure that radiology education aligns with clinical expectations, there is scope to implement structured competency-based assessments. Integrating radiology-focused OSCE stations could provide a consistent and objective means of evaluating students’ ability to interpret images, justify investigations, and communicate findings effectively [15,16]. Moreover, collaboration with national bodies such as the RCR and the General Medical Council (GMC) would help to harmonise standards across UK medical schools, ensuring a consistent baseline of radiological competency upon graduation. Such collaboration could also facilitate the development of national teaching resources and assessment frameworks, promoting sustainability and equity in radiology education. Embedding radiology competence within national outcome frameworks would also strengthen interprofessional learning and clinical readiness [4,5].

Limitations

Several limitations should be acknowledged. First, the narrative literature review was not systematic and therefore may not have captured all relevant studies. Second, the empirical component represents a single-centre pilot evaluation involving a relatively small cohort of Year 3 medical students from one institution, which limits the generalisability of the findings to other medical schools or educational contexts. Third, the study relied primarily on self-reported measures of confidence and perceptions rather than objective assessments of imaging interpretation skills, which may limit the interpretability of the reported improvements. Consequently, improvements observed in this study should be interpreted as increased learner confidence rather than confirmed gains in diagnostic competence. In addition, the survey instrument was developed for the purposes of this educational evaluation and was not formally validated. Fourth, the absence of a control or comparator group prevents direct evaluation of whether case-based teaching is more effective than alternative educational approaches and limits attribution of observed improvements specifically to the intervention. Finally, although participation in the survey was voluntary, its administration during scheduled teaching sessions may have contributed to the high response rate and introduces the potential for response bias. Future research involving multi-institutional cohorts, objective competency assessments, and longitudinal follow-up would help better define the sustained impact of undergraduate radiology teaching interventions.

## Conclusions

Undergraduate radiology education remains variably delivered across institutions and may not consistently align with early postgraduate expectations. The literature review and local pilot findings suggest that gaps may exist in medical students’ understanding of radiology’s clinical and consultative roles, interpretation of common imaging, and awareness of the speciality’s integration within the NHS. Interactive, case-based teaching sessions, designed in line with evidence-based pedagogical principles, significantly improved student confidence and were strongly preferred over traditional lectures, underscoring the value of clinically relevant, hands-on approaches.

Based on our findings, we propose a level-appropriate local curriculum for Year 3 medical students that integrates core imaging skills, clinical case interpretation, and professional awareness of radiology within patient pathways, supported by competency-based frameworks, CBL, integration with clinical practice, and accessible digital resources. This approach aims to bridge the gap between undergraduate education and early postgraduate expectations, equipping students with the knowledge, skills, and confidence required for safe and effective practice as doctors. Future work should focus on longitudinal assessment, multi-institutional implementation, and evaluation of long-term competency retention to inform broader curriculum development.

## Appendices

**Table 10:** Questionnaire used for the pilot radiology teaching evaluation.

Domain	Question	Response Format
Demographics	Year of study	Year 3/Other
Prior exposure	Approximate hours of formal radiology teaching received during the first two years of medical school	0/1-2/3-5/6-10/>10
Prior exposure	What format of radiology teaching have you experienced so far?	Lectures/Small-group teaching/Formal clinical placements/Informal clinical placements/Online learning/None/Other
Prior exposure	Have you received formal teaching in chest radiograph interpretation?	Yes/No
Prior exposure	Have you received formal teaching in CT head interpretation?	Yes/No
Prior exposure	Have you received formal teaching regarding imaging safety and radiation exposure?	Yes/No
Understanding of radiology	In your own words, what do you think radiology is?	Free-text
Understanding of radiology	Do you have an understanding of a radiologist’s day-to-day role?	Clear understanding/Some understanding/Limited understanding/No understanding
Understanding of radiology	Are radiologists routinely involved in multidisciplinary team (MDT) meetings?	Yes/No/Unsure
Understanding of radiology	How important is radiology in patient pathways and clinical decision-making within the NHS?	Very important/Important/Neutral/Limited importance/Unsure
Pre/Post confidence	How confident are you in systematically interpreting a chest radiograph?	1-10 numerical scale
Pre/Post confidence	How confident are you in recognising acute abnormalities on CT head imaging?	1-10 numerical scale
Pre/Post confidence	How confident are you in selecting and justifying appropriate imaging investigations?	1-10 numerical scale
Knowledge & safety	Which imaging modality is associated with the highest radiation dose?	Multiple choice
Knowledge & safety	Which of the following statements regarding ionising radiation is correct?	Multiple choice
Knowledge & safety	Which of the following represents a recognised risk associated with intravenous contrast administration?	Multiple choice
Teaching evaluation (post-session only)	The teaching session improved my confidence in interpreting imaging	5-point Likert scale
Teaching evaluation (post-session only)	Which teaching format do you consider most effective?	Interactive lectures/Case-based teaching/E-learning/Simulation/flipped classroom/Bedside teaching/Other
Teaching evaluation (post-session only)	Should radiology teaching be introduced earlier within the medical curriculum?	Yes/No/Unsure
Teaching evaluation (post-session only)	What aspects of the teaching session were most useful?	Free-text
Teaching evaluation (post-session only)	What improvements would you suggest for future radiology teaching?	Free-text
